# Integrating mental health care into primary and community care in Singapore: a vision based on Healthier SG

**DOI:** 10.1016/j.lanwpc.2024.101279

**Published:** 2024-12-31

**Authors:** Anna Szücs, David C.L. Teo, Jorge Arias De La Torre, Mythily Subramaniam, Jose M. Valderas

**Affiliations:** a Division of Family Medicine, Department of Medicine, Yong Loo Lin School of Medicine, National University of Singapore, Singapore; b Department of Family Medicine, National University Health System, Singapore, Singapore; c Faculty of Behavioural and Movement Sciences, Vrije Universiteit Amsterdam, Netherlands; d Connections MindHealth, Novena Medical Centre, Singapore; e Institute of Psychiatry, Psychology and Neuroscience, King's College London, London, UK; f Care in Long Term Conditions Research Division, King's College London, London, UK; g CIBER Epidemiology and Public Health (CIBERESP), Madrid, Spain; h Institute of Biomedicine (IBIOMED), Universidad de León, León, Spain; i Saw Swee Hock School of Public Health, National University of Singapore, Singapore; j Research Division, Institute of Mental Health, Singapore; k Centre for Research in Health Systems Performance (CRiHSP), National University of Singapore, Singapore, Singapore

Singapore has been considered a world leader of health care system efficiency for the past decade.^1^ Yet, the country has been struggling with effective mental health care delivery, with only about ¼ of mentally ill adults being treated for their condition according to a 2016 population survey.^2^ Healthier SG, as outlined in its September 2022 White Paper,^3^ is an integrated care initiative promoted by the Singaporean government, aimed at reshaping Singapore's healthcare landscape with an emphasis on preventive care and holistic management of chronic conditions, including mental disorders. The main focus of the initiative is to establish collaborative care coordinated by a single primary care provider (PCP) for each Singaporean resident.^3^ Eight months after its launch in July 2023, Healthier SG counted more than 700,000 enrolled residents and over 1,000 PCPs, which encompass both Singapore's public primary care clinics (polyclinics) and private general practitioners (GPs).^4^ Given Healthier SG's broad-ranging nature and the significant means committed by Singapore to its development, the initiative presents an unparalleled opportunity to address long-standing challenges in mental health care and adopt international recommendations of integrated mental health, primary, and community care,^5^ as outlined by Singapore's 2023 National Mental Health and Well-being Strategy.^6^ This commentary aims to provide an overview of Singapore's current mental health care, the reforms envisioned by Healthier SG, and remaining challenges to overcome.

Singapore's current mental health ecosystem possesses multiple resources at the specialised, primary, and community care levels (Fig. 1, upper panel).^7^ Besides inpatient and outpatient mental health care, specialised care services are offering support to primary and community care providers via different expert teams, such as the multidisciplinary Assessment and Shared Care Team (ASCAT), which performs clinical evaluations, short-term follow-ups, and training for PCPs and community care partners. Primary care polyclinics include Health and Mind clinics or Health Wellness clinics led by family physicians in collaboration with ASCAT and other expert teams. While these services are valuable, care remains fragmented due to the complexity of care pathways (Fig. 1, lower panel), difficulties in closed-loop communication between stakeholders, and long waiting times for specialised evaluations.^8^ As a result, primary care services find it challenging to triage referrals and position themselves as an intermediate tier of care between the community and specialist settings. Further, insufficient subsidies and insurance coverage constitute persisting barriers to mental health care for residents.^2^

**Fig. 1:**
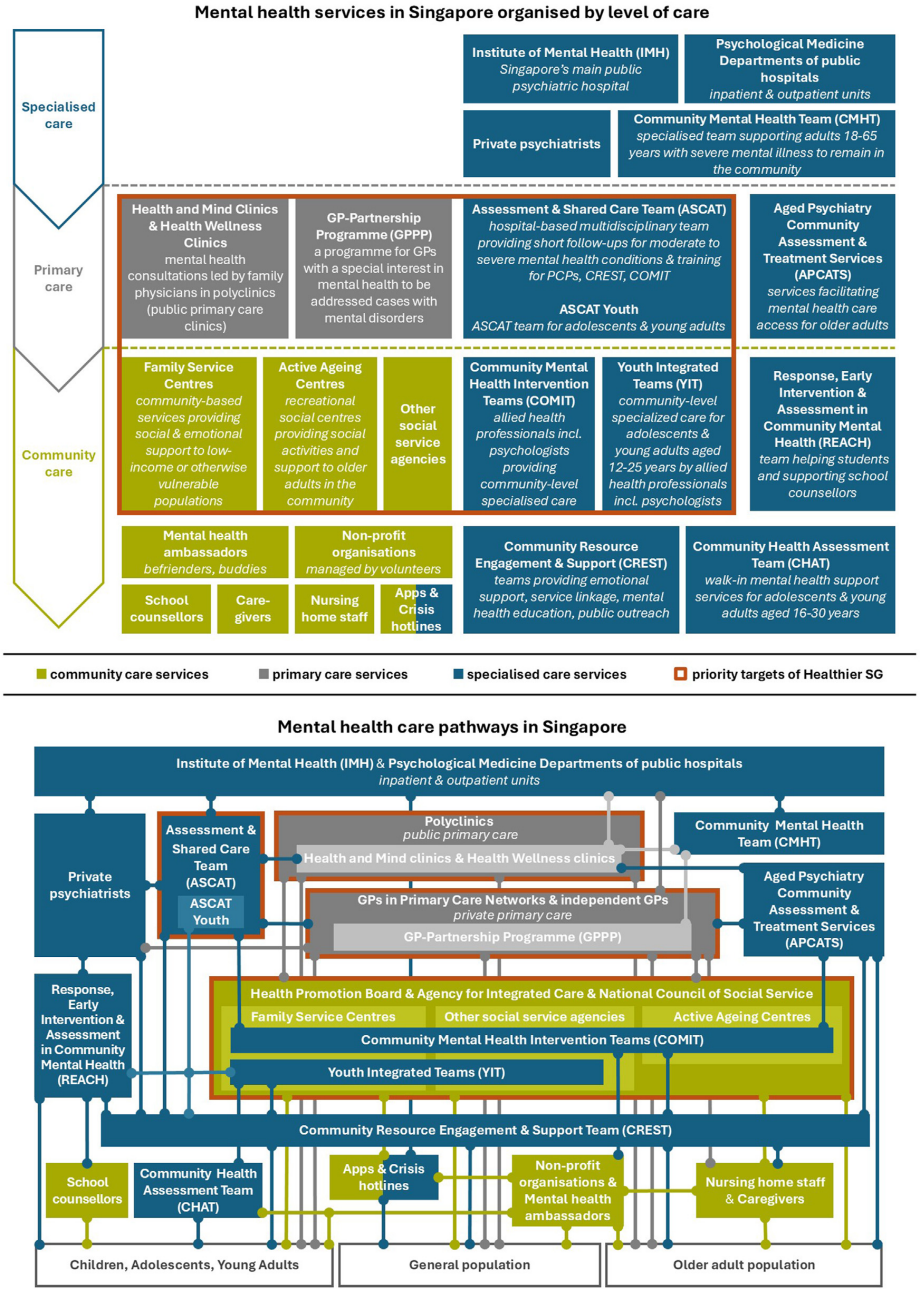
**Organigram of the current mental health care system in Singapore highlighting the services that will be expanded in priority by Healthier SG**. *Note*. Existing mental health care services at the specialised care (blue), primary care (grey), and community care (green) levels in Singapore (upper panel) and main collaboration pathways between them (lower panel). The services outlined in orange will be reinforced in priority by Healthier SG. Apps and crisis hotlines, represented as part of both community and specialised care services in the top panel, encompass resources by various organisations, such as the Institute of Mental Health (crisis hotline), the Samaritans of Singapore (suicide crisis hotline), and Singapore's Health Promotion Board (the *MindSG* app providing self-care resources for mild and moderate mental health conditions). Legend: GP, general practitioner; PCPs, primary care providers from both public and private sectors.

Healthier SG aims to transform this landscape by fostering integrated and cohesive care practices^9^ and making mental health services more affordable by enhancing subsidies through the removal of co-pay requirements for chronic care management. The initiative plans to expand ASCAT to ramp up community access to psychotherapy and mental health care training for PCPs. These approaches have shown improvement in mental health care availability, including for medically underserved populations.^10^^,^^11^ In turn, PCPs will be incentivised to deliver preventive care and manage milder mental health cases at their level. The regular health check-ins and personalised health plans encouraged by Healthier SG will further enable early intervention for possible mental disorders by engaging patients at each stage of the care process and thus improving their mental health literacy, awareness, and self-management competency. Besides, through the development of an enhanced information technology infrastructure and data sharing of medical health records, Healthier SG will facilitate the coordination of care between PCPs and other care providers. Resulting data analytics may be used at the population's level to monitor effectiveness and inform subsequent health policies.^12^ Finally, Singapore's community organisations, the Health Promotion Board and Agency for Integrated Care, will receive means to enhance the overall mental health of the population by providing a wider range of personalised social support services, outreach programmes, and neighbourhood activities promoting healthy living, social cohesion, and wellbeing.

There nonetheless remain unaddressed challenges. Mental health stigma^13^ and local cultural values (e.g., esteeming self-control over emotional sharing)^8^ may require to individualise mental health care in a culturally sensitive manner. GPs may experience the steepest increase in their workload, as they will need to manage a larger proportion of multimorbid patients without all the team-based resources available to polyclinics. Yet, GPs' confidence and willingness to treat mental health conditions such as depression varies greatly.^8^ Lastly, measuring quality and key performance indicators to base remunerations on may require mixed, quantitative-qualitative approaches for mental health, not generally employed with somatic conditions.^14^ To achieve the necessary shifts in practices over time, health system leadership needs to focus on engaging, educating, and supporting all stakeholders including GPs, community partners, and patients. It may draw inspiration from promising primary mental health care reforms implemented elsewhere, including Australia's Mental Health Professionals Network,^15^ which has been successful at catalysing collaborative practice changes among GPs.^16^ However, to effectively transpose such reforms to the Singaporean context, the interplay between system-level factors, such as time constraints and remuneration, and provider attitudes should be better understood locally, through more qualitative research on primary mental health care.

In conclusion, Healthier SG represents a comprehensive and ambitious plan to enhance Singapore's healthcare system, with significant implications for community mental health provision. Its proposal of integrating mental health care into routine preventive care coordinated by PCPs, with whom patients have already built rapport, can encourage help-seeking and early intervention, while fostering a supportive environment for mental well-being in general. Tightening collaboration between PCPs, mental health specialists, and community partners will be paramount in achieving care continuity and closed-loop communication, in turn fundamental to this initiative's long-term goals, namely improving mental health care access and utilisation and, ultimately, alleviating the burden of mental disorders in Singapore.

## Contributors

**Anna Szücs**: conceptualisation, visualisation, writing–original draft; **David C L Teo**: conceptualisation, writing–review & editing; **Jorge Arias De La Torre**: conceptualisation, writing–review & editing; **Mythily Subramaniam**: conceptualisation, writing–review & editing; **Jose M Valderas**: conceptualisation, funding acquisition, writing–original draft.

## Declaration of interests

The authors have no conflicting interests to declare.
